# Guided Relaxation–Based Virtual Reality for Acute Postoperative Pain and Anxiety in a Pediatric Population: Pilot Observational Study

**DOI:** 10.2196/26328

**Published:** 2021-07-12

**Authors:** Vanessa A Olbrecht, Keith T O'Conor, Sara E Williams, Chloe O Boehmer, Gilbert W Marchant, Susan M Glynn, Kristie J Geisler, Lili Ding, Gang Yang, Christopher D King

**Affiliations:** 1 Center for Understanding Pediatric Pain Department of Anesthesiology Cincinnati Children's Hospital Medical Center Cincinnati, OH United States; 2 Department of Anesthesiology Cincinnati Children's Hospital Medical Center Cincinnati, OH United States; 3 Center for Understanding Pediatric Pain Division of Behavioral Medicine and Clinical Psychology, Department of Pediatrics Cincinnati Children's Hospital Medical Center Cincinnati, OH United States; 4 Division of Biostatistics and Epidemiology Department of Pediatrics Cincinnati Children's Hospital Medical Center Cincinnati, OH United States

**Keywords:** virtual reality, guided relaxation–based virtual reality, pain, anxiety, acute pain, postoperative pain, pediatrics

## Abstract

**Background:**

Distraction-based therapies, such as virtual reality (VR), have been used to reduce pain during acutely painful procedures. However, distraction alone cannot produce prolonged pain reduction to manage sustained postoperative pain. Therefore, the integration of VR with other pain-reducing therapies, like guided relaxation, may enhance its clinical impact.

**Objective:**

The goal of this pilot study was to assess the impact of a single guided relaxation–based VR (VR-GR) session on postoperative pain and anxiety reduction in children. We also explored the influence of pain catastrophizing and anxiety sensitivity on this association.

**Methods:**

A total of 51 children and adolescents (7-21 years) with postoperative pain and followed by the Acute Pain Service at Cincinnati Children’s Hospital were recruited over an 8-month period to undergo a single VR-GR session. Prior to VR, the patients completed 2 questionnaires: Pain Catastrophizing Scale for Children (PCS-C) and the Child Anxiety Sensitivity Index (CASI). The primary outcome was a change in pain intensity following the VR-GR session (immediately, 15 minutes, and 30 minutes). The secondary outcomes included changes in pain unpleasantness and anxiety.

**Results:**

The VR-GR decreased pain intensity immediately (*P*<.001) and at 30 minutes (*P*=.04) after the VR session, but not at 15 minutes (*P*=.16) postsession. Reductions in pain unpleasantness were observed at all time intervals (*P*<.001 at all intervals). Anxiety was reduced immediately (*P*=.02) but not at 15 minutes (*P*=.08) or 30 minutes (*P*=.30) following VR-GR. Patients with higher CASI scores reported greater reductions in pain intensity (*P*=.04) and unpleasantness (*P*=.01) following VR-GR. Pain catastrophizing was not associated with changes in pain and anxiety.

**Conclusions:**

A single, short VR-GR session showed transient reductions in pain intensity, pain unpleasantness, and anxiety in children and adolescents with acute postoperative pain. The results call for a future randomized controlled trial to assess the efficacy of VR-GR.

**Trial Registration:**

ClinicalTrials.gov NCT04556747; https://clinicaltrials.gov/ct2/show/NCT04556747

## Introduction

Ineffective postoperative pain management, defined as surgery-related pain resulting from tissue injury and muscle spasm, has long-term consequences, including increased morbidity, poorer physical functioning, longer recovery, and increased costs [1]. Despite the widespread use of multimodal analgesia, pediatric postoperative pain remains difficult to manage [2], increasing the risk of persistent postoperative pain [3]. Studies of pediatric postoperative patients identified a 20% incidence of persistent pain beyond that expected from surgery [4]. While 80% of the patients included in this study recovered within 1 month, 20% reported reduced quality of life [4].

Although multimodal analgesia protocols focus on regional analgesia and nonopioid medications, opioid usage remains ubiquitous in pain management. Children and adolescents are at particular risk of long-term opioid abuse—as few as 5 days of use increases this risk [5]. A study of opioid-naïve pediatric surgical patients found persistent opioid use in 4.8% of adolescents versus 0.1% in a matched, nonsurgical cohort [3]. Other studies found that more than 25% of patients transitioned to chronic opioid consumption [6].

There remains a critical need for novel, nonpharmacologic pain management strategies, like virtual reality (VR). VR provides an immersive, multisensory, 3D environment that modifies the experiences of reality and creates a “sense of presence.” This “sense of presence” makes VR an excellent distraction-based therapy [7]. The use of distraction–based VR (VR-D) has been used to reduce pain in acute procedural, postoperative, and labor pain management by redirecting attention (eg, distraction) [8-14]. These transient reductions are sufficient for short-term reductions in pain, but they are not sufficient to treat prolonged acute pain experiences [15,16], including postoperative pain.

Alternative interventions utilizing traditionally delivered mind body–based therapies, like relaxation and slow breathing, reduce anxiety and pain in children undergoing surgery [17]. Combining these traditional mind-body therapies with VR, like guided relaxation–based VR (VR-GR), opens new possibilities for multimodal analgesia that may impact postoperative pain management and elevates VR therapy beyond simple distraction alone.

This study aimed to pilot the use of a single session of VR-GR in children after surgery to assess the association between VR-GR and pain intensity in addition to exploring the effect of pain catastrophizing and anxiety sensitivity on this association. We further assessed the association between VR-GR and reductions in pain unpleasantness and anxiety. We hypothesized that one VR-GR session would be associated with transient reductions in pain intensity, pain unpleasantness, and anxiety, with the greatest within-patient changes in children having high baseline pain catastrophizing and anxiety sensitivity.

## Methods

**Figure 1:**
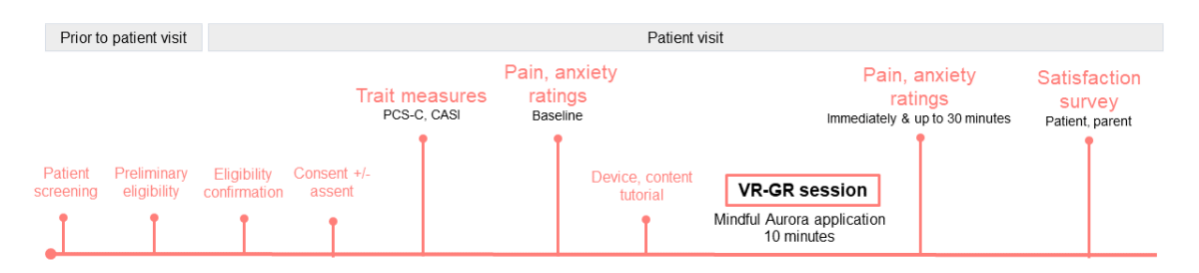
Study diagram. CASI: Child Anxiety Sensitivity Index; PCS-C: Pain Catastrophizing Scale for Children; VR-GR: guided relaxation–based virtual reality.

A single-center, prospective, pilot study in a broad pediatric postoperative population experiencing moderate to severe pain was designed to preliminarily assess the correlation between a single VR-GR session and reductions in acute postoperative pain and anxiety, to explore the association between these within-patient changes and pain catastrophizing and anxiety sensitivity, and to show the feasibility of using this technology in a postoperative setting (Figure 1). Additionally, the study results will assist in developing a randomized controlled trial comparing the efficacy of VR-GR to active control, and will help in providing the preliminary effect data to assist with sample size determination.

### Patients

The study recruited 51 children and adolescents who underwent surgery and were followed by the Acute Pain Service at Cincinnati Children’s Hospital Medical Center between July 2019 and March 2020. The study was approved by the Institutional Review Board (IRB #2018-2892) and conducted per the rules and regulations for ethical research. It was registered with ClinicalTrials.gov on September 21, 2020 (NCT04556747). A written consent (and assent for patients >11 years) was obtained from all participants and the study adhered to the CONSORT (Consolidated Standards of Reporting Trials) guidelines. The patients were recruited and enrolled in the study after surgery following hospital admission, and they were not compensated for participation.

Patients were suitable for recruitment if the Acute Pain Service was managing their pain. The patients managed by the Acute Pain Service experience high postoperative pain unmanageable solely by the surgery team. These patients necessitate multimodal analgesia and management with patient-controlled analgesia (PCA) and regional or neuraxial analgesia.

#### Inclusion Criteria

The eligibility criteria for inclusion were: 7-21 years old; able to read, speak, and write English; and undergone surgery resulting in significant postoperative pain necessitating care by the Acute Pain Service.

#### Exclusion Criteria

Patients were excluded if they did not fall within the age criteria; unable to read, speak, or write English; had a history of developmental delay, neurological conditions (seizure disorder, vertigo, dizziness, or significant motion sickness/nausea/vomiting), or uncontrolled psychiatric conditions; or had head or neck surgery that precluded the use of VR.

#### Patient Information

Prior to the VR-GR session, we collected the patient’s age, sex, race, surgery type, and American Society of Anesthesiologists (ASA) status. The ASA status is a 6-category classification system that assesses a patient’s general health before surgery; the sickness of a patient increases with the category number. Class I and II are healthy or have mild/moderate systemic disease; class III and IV patients have severe disease that may limit activity or threaten life.

### Measures

The primary outcome was the change in pain intensity after VR-GR. Secondary outcome measures included changes in pain unpleasantness and anxiety.

#### Pain Intensity, Pain Unpleasantness, and Anxiety

Pain intensity, pain unpleasantness, and anxiety were measured before and after the VR-GR sessions using the numerical rating scale (NRS) [18]. A script was used to explain the difference between pain intensity and pain unpleasantness. Pain was described as analogous to listening to music on the radio: pain intensity as the music volume and pain unpleasantness as how much the patient disliked the music [19]. The patients rated symptom severity from 0 to 10 (0=nonexistent, 10=most severe) for each measure.

#### Pain Catastrophizing

The Pain Catastrophizing Scale for Children (PCS-C), a validated, self-reported questionnaire assessing pain catastrophizing tendencies [20,21], was completed before the VR-GR session. The survey was completed on an iPad by the patient directly into a REDCap database. All but one patient, who required that the survey be read to him or her, completed the survey independently. The patients used a 5-point Likert scale to rate 13 items assessing rumination, magnification, and helplessness related to thoughts about pain. The summary scores were interpreted as low=0-14, moderate=15-25, and high >26 [21]. Internal reliability in the sample was high (Cronbach α=.92).

#### Anxiety Sensitivity

The Child Anxiety Sensitivity Index (CASI) questionnaire was completed before the VR-GR as described above. The CASI has been used in VR studies in adolescents aged 10-21 years [12], validated in children, and is an 18-item survey that measures how patients perceive anxiety symptoms [22]. The total scores range from 18-54 [22], and the sample’s internal reliability was good (Cronbach α=.86). Both surveys took less than 5 minutes to complete.

#### Patient Experience

Patient experience was measured with the study team–generated questionnaire. The participants ranked how much they agreed with statements on a 4-point Likert scale. The parent(s) of the participants were asked to fill out a similar survey to understand their perspective of the VR experience. The surveys, included as Multimedia Appendices 1 and 2, were completed independently on an iPad and entered directly into REDCap; 1 patient required that the survey be read aloud for completion. The surveys took less than 5 minutes to complete.

### VR Device and Content

**Figure 2:**
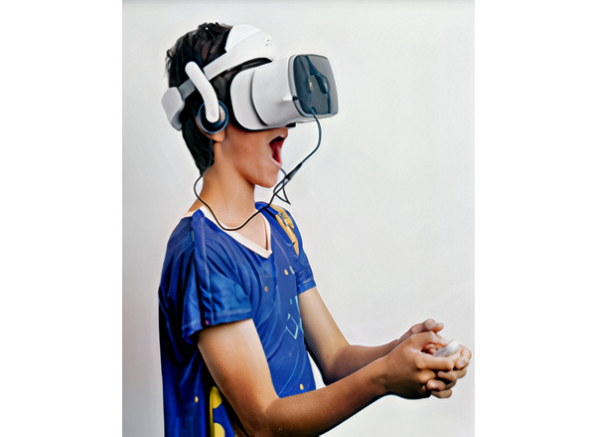
A child wearing a Starlight Xperience headset.

**Figure 3:**
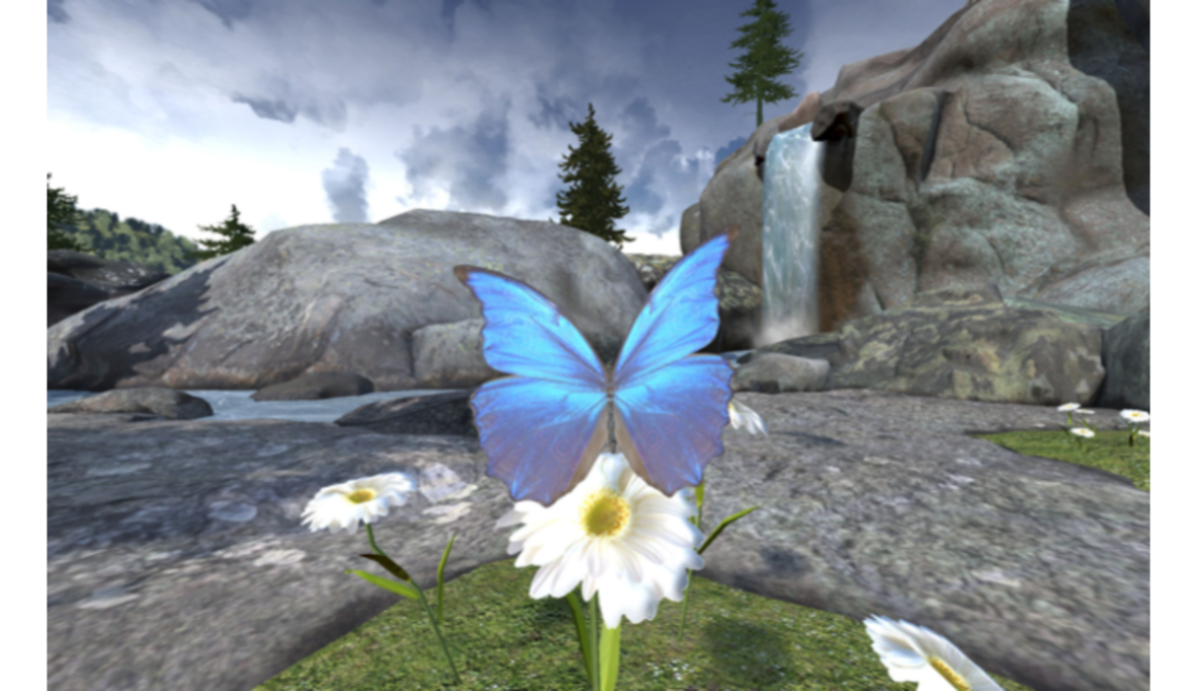
A snapshot of the Mindful Aurora application.

All patients used the Starlight Xperience VR device, a commercially available headset supplied by the Starlight Children’s Foundation (Figure 2). It is a customized version of the Lenovo Mirage Solo with Daydream VR headset. The integrated headphones deliver audio content, and the patients interact and navigate within the VR environment using head movements and a handheld controller. The patients used the “Mindful Aurora” guided relaxation–based application to learn slow breathing and relaxation, an application developed by the Stanford CHARIOT (Childhood Anxiety Reduction through Innovation and Technology) program (Figure 3). The users were “transported” to an alpine meadow in the virtual world, where a 10-minute relaxation narrative teaches focused, slow, and paced breathing.

### Procedures

The patient visits occurred on postoperative day (POD) 1 or 2. After determining eligibility and obtaining consent, the patients completed the PCS-C and CASI questionnaires and rated their baseline pain intensity, pain unpleasantness, and anxiety levels on the NRS.

Following these assessments, the patients were oriented to the VR headset and given a tutorial on the device application. They were instructed to remove the headset for discomfort, nausea, or dizziness. After completing the 10-minute session (based on the standard duration of relaxation sessions), the VR device was removed. Pain intensity, pain unpleasantness, and anxiety were recorded immediately, at 15 minutes, and at 30 minutes after the experience. The patients and parents then completed the experience questionnaires.

### Statistical Analysis

All statistical analyses were performed using SAS 9.4 (SAS Institute). A *P* value of .05 was the cutoff value for statistical significance. Additionally, statistical significance with the Bonferroni adjustment for multiple comparisons for the primary outcome (change from the baseline pain intensity at three time-points after VR-GR) was assessed. The first-order autoregressive, AR(1), covariance structure was used in all the mixed-effects models. The missing data were examined, and all available data were used in statistical analyses.

#### Descriptive Analysis

Descriptive statistics were calculated for all baseline variables and change from the baseline for outcome variables. Mean (SD) and median (IQR) were used for the continuous variables, while frequency and percentage were used for the categorical variables.

#### Changes in Pain and Anxiety Following VR-GR

Pain intensity, unpleasantness, and anxiety after the VR-GR session were compared to the baseline within each patient using paired tests (*t* test or signed-rank, as appropriate) at individual time points. The changes from baseline were analyzed with mixed-effects models where time intervals (immediately, 15 minutes, or 30 minutes after VR-GR), pain catastrophizing, and anxiety sensitivity were used as the categorical fixed effects.

#### Associations in Baseline Outcomes

The Pearson or Spearman correlation coefficients were derived to test the association between the traits (pain catastrophizing and anxiety sensitivity) and outcomes (pain intensity, pain unpleasantness, and anxiety).

#### Impact of Psychological Factors on Changes in Pain and Anxiety

Mixed-effects models were used to examine the association of PCS-C and CASI on the changes from the baseline of pain intensity, pain unpleasantness, and anxiety, where time intervals (immediately, 15 minutes, or 30 minutes after the VR-GR) were used as the categorical fixed effect.

## Results

### Participant Characteristics

We enrolled 51 patients over 8 months. All patients completed pain and anxiety assessments at baseline and immediately following VR-GR; 100% (n=51) completed the pain assessment at 15 minutes, and 98% (n=50) completed the anxiety assessment at 15 minutes; and 88% (n=45) completed the assessments at 30 minutes following VR-GR. The patients did not receive any analgesic medications during participation in the study. Missing data resulted from limitations in the clinical environment, including patients undergoing imaging studies, receiving care from the care team, or falling asleep.

The patients were primarily adolescent, male, and Caucasian (Table 1). Of the 51 patients recruited, 19 (37.3%) underwent abdominal surgery, 21 (41.2%) underwent Nuss repair of pectus excavatum or chest surgery, and 11 (21.6%) underwent orthopedic procedures (such as posterior spinal fusion or major hip surgery). Half of the recruited patients were ASA status I/II or III/IV. The patients reported moderate pain intensity, unpleasantness, and mild anxiety levels before the VR-GR (Table 1).

Patients had moderate pain catastrophizing and average anxiety sensitivity [21]. Furthermore, higher PCS-C scores were associated with higher baseline anxiety (Spearman ρ=0.41, *P*<.001) (Table 2).

Pain catastrophizing and anxiety sensitivity were not associated with changes in pain intensity or pain unpleasantness.

**Table 1:** Demographic, survey, and medical data from study participants (N=51).

Variable		Value
Age (years)		14.6 (3.2)
**Sex, n (%)**		
	Male	32 (63)
	Female	19 (37)
**Race, n (%)**		
	Caucasian	41 (80)
	Non-Caucasian	10 (20)
**Surgery type, n (%)**		
	Pectus/chest	21 (41)
	Abdominal	19 (37)
	Orthopedic	11 (22)
**ASA^a^** **physical status, n (%)**		
	I/II (healthy, mild systemic disease)	24 (47)
	III/IV (severe or life-threatening disease)	24 (47)
**Baseline NRS^b^** **scores, n (%)**		
	Pain intensity (0**-**10)	5.11 (1.74)
	Pain unpleasantness (0**-**10)	5.73 (2.30)
	Anxiety (0**-**10)	2.05 (2.50)
**Psychological factors, n (%)**		
	Catastrophizing (PCS-C^c^)	21.6 (11.0)
	Anxiety sensitivity (CASI^d^)	31.2 (3.9)

^a^ASA: American Society of Anesthesiologists; ASA status was not collected on three (n=3) patients.

^b^NRS: numerical rating scale.

^c^PCS-C: Pain Catastrophizing Scale for Children.

^d^CASI: Child Anxiety Sensitivity Index.

**Table 2:** The Spearman correlation between baseline numerical rating scale (NRS) scores and surveys.

Baseline NRS	Pain catastrophizing (PCS-C^a^)	*P* value	Anxiety sensitivity (CASI^b^)	*P* value
Pain intensity	0.19	.17	–0.09	.55
Pain unpleasantness	0.17	.23	0.06	.67
Anxiety	0.41	.003	0.25	.07

^a^PCS-C: Pain Catastrophizing Scale for Children.

^b^CASI: Child Anxiety Sensitivity Index.

### Primary Outcome: Pain Intensity

**Figure 4:**
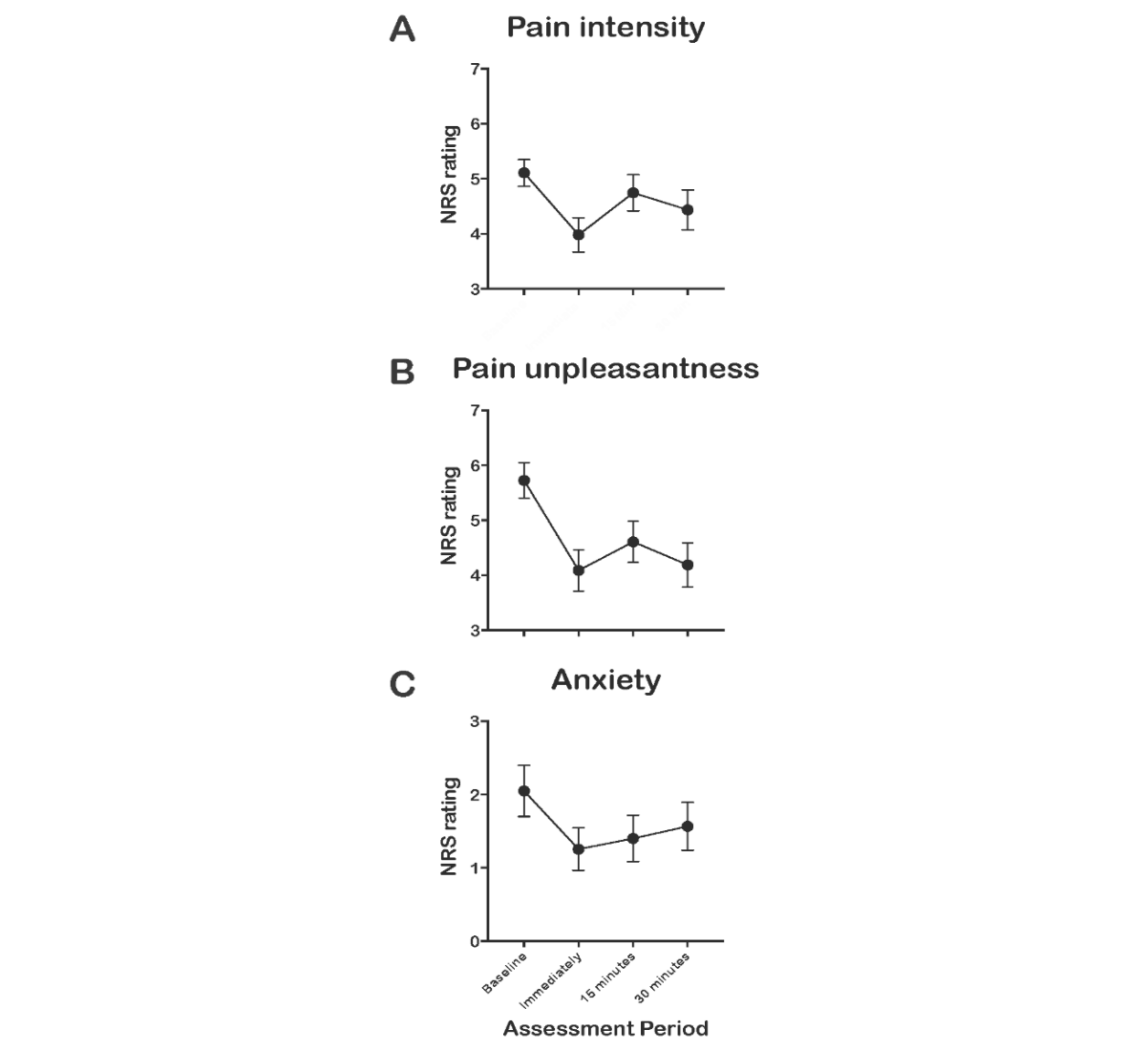
Changes in pain intensity, pain unpleasantness, and anxiety numerical rating scale (NRS) scores at baseline, immediately, 15 minutes, and 30 minutes. (A) The Wilcoxon signed-rank test showing the VR-GR correlation with pain intensity: immediately (median –1.0, IQR –2.0 to 0, *P*<.001); at 15 minutes (median 0, IQR –1.0 to 0.50, *P*=.03); at 30 minutes (median 0, IQR –1.5 to 0, *P*=.02). (B) The Wilcoxon signed-rank test showing the VR-GR correlation with pain unpleasantness: immediately (median –2.0, IQR –3.0 to 0, *P*<.001); at 15 minutes (median –1.0, IQR –2.0 to 0, *P*<.001); and at 30 minutes (median –1.0, IQR –3.0 to 0, *P*<.001). (C) The Wilcoxon signed-rank test showing an association between the VR-GR and anxiety: immediately (median 0, IQR –1.0 to 0, *P*<.001); at 15 minutes (median 0, IQR –1.0 to 0, *P*=.01); at 30 minutes (median 0, IQR –1.0 to 0, *P*=.17).

VR-GR was associated with a small reduction in pain intensity (Figure 4A, Table 3). The Wilcoxon signed-rank test showed that pain intensity decreased immediately (median –1.0, IQR –2.0 to 0, *P*<.001) following the VR-GR session and remained significant at 15 minutes (median 0, IQR –1.0 to 0.50, *P*=.03), and at 30 minutes (median 0, IQR –1.5 to 0, *P*=.02) (Table 3).

**Table 3:** Mean (SD) and median (IQR) changes in pain intensity, pain unpleasantness, and anxiety numerical rating scale (NRS) scores following the guided relaxation–based virtual reality (VR-GR). The Wilcoxon signed-rank test was used to compare the changes.

NRS outcome following the VR-GR			N		Mean (SD)	Median (IQR)	*P* value
**Pain intensity**							
	Immediately	51		–1.1 (1.1)		–1 (–2.0 to 0)	<.001
	15 minutes	51		–0.4 (1.5)		0 (–1.0 to 0.5)	.03
	30 minutes	45		–0.5 (1.8)		0 (–1.5 to 0)	.02
**Pain unpleasantness**							
	Immediately	51		–1.6 (1.6)		–2 (–3.0 to 0)	<.001
	15 minutes	51		–1.1 (1.9)		–1 (–2.0 to 0)	<.001
	30 minutes	45		–1.4 (1.9)		–1 (–3.0 to 0)	<.001
**Anxiety**							
	Immediately	51		–0.8 (1.4)		0 (–1.0 to 0)	<.001
	15 minutes	51		–0.6 (1.7)		0 (–1.0 to 0)	.01
	30 minutes	45		–0.4 (1.7)		0 (–1.0 to 0)	.17

### Secondary Outcomes

#### Pain Unpleasantness

VR-GR was also associated with a change in pain unpleasantness (Figure 4B). The Wilcoxon signed-rank test (Table 3) revealed that pain unpleasantness decreased immediately following VR-GR (median –2.0, IQR –3.0 to 0, *P*<.001), and remained significantly decreased at 15 minutes (median –1.0, IQR –2.0 to 0, *P*<.001), and at 30 minutes (median –1.0, IQR –3.0 to 0, *P*<.001) following VR-GR.

#### Anxiety

We also found an association between VR-GR and anxiety (Figure 4C). The Wilcoxon signed-rank test (Table 3) revealed anxiety decreased from the baseline to immediately following VR-GR (median 0, IQR –1.0 to 0, *P*<.001); it remained lower at 15 minutes (median 0, IQR –1.0 to 0, *P*<.001) but not at 30 minutes.

### Association Between Psychological Factors and Changes in Outcomes From the Baseline

We used a series of adjusted, mixed-effects models to observe the correlation between psychological factors and changes in the baseline outcomes. It was observed that anxiety sensitivity levels were associated with greater reductions in pain intensity (β=–.06, SE 0.03, *P*=.04) and pain unpleasantness (β=–.09, SE 0.04, *P*=.01). Pain catastrophizing was not associated with changes in pain and anxiety following VR-GR (Table 4).

**Table 4:** Association between psychological factors and changes in pain intensity, pain unpleasantness, and anxiety numerical rating scale (NRS) scores following the guided relaxation–based virtual reality (VR-GR).

Change from the baseline following the VR-GR			Beta (SE)		*P* value
**Pain intensity**					
	Pain catastrophizing (PCS-C^a^)	–.0001 (0.01)		.99	
	Anxiety sensitivity (CASI^b^)	–.04 (0.02)		.06	
**Pain unpleasantness**					
	Pain catastrophizing (PCS-C)	.01 (0.02)		.50	
	Anxiety sensitivity (CASI)	–.05 (0.03)		.08	
**Anxiety**					
	Pain catastrophizing (PCS-C)	–.02 (0.02)		.25	
	Anxiety sensitivity (CASI)	–.06 (0.03)		.04	

^a^PCS-C: Pain Catastrophizing Scale for Children.

^b^CASI: Child Anxiety Sensitivity Index.

When assessing the association between changes from the baseline and covariates, we found that the Caucasian patients had a smaller decrease in pain unpleasantness from the baseline than the non-Caucasian participants—difference in least squares mean (LSM=1.20, 95% CI 0.11-2.28, *P*=.03). Additionally, the older participants had a smaller decrease in pain intensity (β=.12, SE 0.05, *P*=.03) than the younger patients. No other variables (ASA, POD, or race) were associated with changes in outcomes.

### Satisfaction With VR

The patients reported a very positive VR experience. Overall, 96% of children would recommend VR to friends and family, and most (n=45, 88%) believed they felt “calmer and less anxious after having used VR” and that VR “made it easier for (them) to tolerate (their) pain.” Parents reported a similar positive overall experience when asked the same questions. The parents (n=44, 100%) who completed the questionnaire would recommend VR; 93% (n=41) believed that VR made their child calm, and 84% (n=37) believed that VR helped their child tolerate pain. No patient experienced any self-reported side effects during the VR session.

## Discussion

### Principal Findings

VR-GR may be beneficial in pediatric postoperative pain management. Our pilot study assessed the association between a single VR-GR session delivered after surgery and changes in pain intensity, pain unpleasantness, and anxiety in children and adolescents with acute pain following surgery. The most significant changes were observed for pain unpleasantness, with lesser reductions in pain intensity and anxiety. More considerable reductions in pain intensity and unpleasantness after VR-GR were associated with higher anxiety sensitivity. Pain catastrophizing did not appear to be associated with changes in these outcomes. The qualitative assessments suggested that the patients had positive experiences with VR-GR with no reported side effects.

Although pain management strategies increasingly incorporate multimodal analgesia, the percentage of patients experiencing severe postoperative pain did not change in the last 20 years [2]. Besides prolonged pain, children are also at risk of persistent opioid use after surgery; opioid exposure after surgery can confer nearly a 50-fold increase in opioid use [3]. It was reported that more than 25% of children with chronic pain transitioned into chronic opioid use after using opioids for surgery-associated pain [6].

Few studies have used VR for acute postoperative pain in adults [23], and none have used it to help manage pain in children after surgery. Most VR studies use distraction–based VR (VR-D) to reduce pain temporarily by redirecting attention [7,9,10,13]. Without VR, distraction alone provides little pain management benefit [24], with no significant lasting impact on pain [15]. The improved efficacy of VR-D to temporarily reduce pain versus distraction alone may be due to the immersion with VR [23]. Although VR-D is more effective than distraction alone, its use is limited. Incorporating VR with other pain-reducing strategies, like guided relaxation, may promote sustained pain relief beyond the temporary impact of distraction [16].

Mind-body therapies, like relaxation and guided imagery, decrease anxiety and help manage pain in children undergoing surgery [17]. Unfortunately, their integration into postoperative clinical care is fraught with challenges, including access to care, high cost, and provider availability. As a result, the use of these therapies is minimal even at a tertiary care institution like ours, besides their inability to engage children. Using VR to deliver these therapies can increase accessibility and enhance acceptability in children versus methods without VR, making this therapy more engaging and relevant. Importantly, VR and mind-body therapies have very little side effects, a significant advantage over traditional pharmacologic interventions.

This study was the first to integrate a mind-body therapy with VR to help pediatric postoperative pain. The study’s goals were to preliminarily assess the use of VR-GR to decrease acute postoperative pain and anxiety and determine whether pain catastrophizing and anxiety sensitivity influence the VR-GR effect in a broad pediatric population. The “Mindful Aurora” application taught patients to slow down their breathing and relax. A single VR-GR session was associated with immediate, acute changes in pain intensity, pain unpleasantness, and anxiety. Reductions in pain unpleasantness were observed at all intervals (immediately, 15 minutes, and 30 minutes following VR-GR). Anxiety reduced immediately and at 15 minutes following VR-GR. An association was observed between higher CASI scores and greater reductions in pain intensity and unpleasantness following VR-GR; however, such an association was absent with pain catastrophizing.

### Limitations

This study had several limitations. The study design did not include a control group, which restricted us from determining a causal relationship between the use of VR-GR and pain and anxiety reduction. It is possible that interaction with the study team could influence changes in pain and anxiety. Additionally, we did not collect data on analgesic use in the study population or standardize the timing of the postoperative visit. This study was designed to test the feasibility of the VR-GR technology, obtain pilot data in children hospitalized after surgery, and assess a possible association between VR-GR and pain and anxiety reduction. The results of this study support the need to conduct a randomized controlled efficacy trial comparing the use of VR-GR to active control, data collection on the timing and use of all analgesic medications, and standardizing the timing of the VR-GR sessions. The study used a single, short VR session in the postoperative period. Although we saw small changes in pain intensity, pain unpleasantness, and anxiety, these changes did not reach any clinical significance. Previous literature report that a reduction in pain intensity of 2 or more points on the NRS or a 30% reduction in pain is considered clinically significant [25]. We recognize that a single session is unlikely to produce a large and sustained effect, and we will pursue further clinical studies using repeated sessions to determine the impact on these outcomes. Finally, the potential bias when self-reporting pain scores could have produced lower self-reported pain and anxiety scores following the VR-GR session. This will be addressed in future studies by including a control group.

### Conclusions

To summarize, our study demonstrates the successful use of VR-GR in children after surgery. A single VR-GR session was associated with transient decreases in pain and anxiety. Future research is needed to investigate the effect of VR-GR in reducing pain and anxiety in the postoperative setting compared to a control.

## Appendix

Multimedia Appendix 1 Patient experience questionnaire - parent.

Multimedia Appendix 2 Patient experience questionnaire - child.

## Abbreviations

ASA: American Society of Anesthesiologists
CASI: Child Anxiety Sensitivity Index
CONSORT: Consolidated Standards of Reporting Trials
CHARIOT: Childhood Anxiety Reduction through Innovation and Technology
LSM: least squares mean
NRS: numerical rating scale
PCA: patient-controlled analgesia
PCS-C: Pain Catastrophizing Scale for Children
POD: postoperative day
VR: virtual reality
VR-D: distraction–based virtual reality
VR-GR: guided relaxation–based virtual reality
