# Supplementary material for: Guided Relaxation–Based Virtual Reality for Acute Postoperative Pain and Anxiety in a Pediatric Population: Pilot Observational Study
Source: J Med Internet Res. 2021 Jul 12;23(7):e26328. doi: 10.2196/26328 (PMC8314162; doi:10.2196/26328)
Supplement: Multimedia Appendix 1 [file jmir_v23i7e26328_app1.pdf]

Patient Experience Questionnaire - Parent (PEQ-P)

Please complete the survey below.

Thank you!

Please mark the extent to which you agree or disagree with the following statements:

		Strongly Agree	Agree	Disagree	Strongly Disagree
1)	The instructions that my child received prior his or her virtual reality therapy were helpful and adequate.	◯	◯	◯	◯
2)	The equipment worked well without any bugs or malfunctions.	◯	◯	◯	◯
3)	I would recommend friends or family to try virtual reality during their visits at the clinic.	◯	◯	◯	◯
4)	My child felt calmer and less anxious after having used virtual reality.	◯	◯	◯	◯
5)	Virtual reality made it easier for my child to tolerate his or her procedure(s).	◯	◯	◯	◯
6)	Virtual reality made it easier for my child to tolerate his or her pain.	◯	◯	◯	◯

7) What is your current knowledge about computers?

◯ None
◯ Basic
◯ Intermediate
◯ Expert

8) How often do you play video games?

◯ Never
◯ Occasionally (once or twice a month)
◯ Frequently, but less than 50% of the days
◯ Frequently, 50% of the days or more

9) What is your current knowledge about VR?

◯ None
◯ Basic
◯ Intermediate
◯ Expert
