# Supplementary material for: Guided Relaxation–Based Virtual Reality for Acute Postoperative Pain and Anxiety in a Pediatric Population: Pilot Observational Study
Source: J Med Internet Res. 2021 Jul 12;23(7):e26328. doi: 10.2196/26328 (PMC8314162; doi:10.2196/26328)
Supplement: Multimedia Appendix 2 [file jmir_v23i7e26328_app2.pdf]

Patient Experience Questionnaire - Child (PEQ-C)

Please complete the survey below.

Thank you!

Please mark the extent to which you agree or disagree with the following statements:

		Strongly Agree	Agree	Disagree	Strongly Disagree
1)	The instructions I received prior my virtual reality therapy were helpful and adequate.	○	○	○	○
2)	The equipment worked well without any bugs or malfunctions.	○	○	○	○
3)	I would recommend friends or family to try virtual reality during their visits at the clinic.	○	○	○	○
4)	I felt calmer and less anxious after having used virtual reality.	○	○	○	○
5)	Virtual reality made it easier for me to tolerate my procedure(s).	○	○	○	○
6)	Virtual reality made it easier for me to tolerate my pain.	○	○	○	○

Please indicate how much you agree or disagree with each of the following statements by selecting one of the numbers on the scale below. After my experience of the displayed environment...

		Strongly Disagree	Somewhat Disagree	Neither Agree of Disagree	Somewhat Agree	Strongly Agree
7)	I had a sense that I returned from a journey.	○	○	○	○	○
8)	I vividly remember some parts of the experience.	○	○	○	○	○
9)	I felt myself being 'drawn in.'	○	○	○	○	○
10)	I felt involved.	○	○	○	○	○
11)	I lost track of time.	○	○	○	○	○
12)	I felt that the characters and/or objects could almost touch me.	○	○	○	○	○
13)	I felt I was visiting the places in the displayed environment.	○	○	○	○	○

14)

	I felt that the displayed environment was part of the real world.	◯	◯	◯	◯	◯
15)	My experience was intense.	◯	◯	◯	◯	◯
16)	I paid more attention to the displayed environment than I did to my own thoughts.	◯	◯	◯	◯	◯

PLEASE ANSWER THE NEXT FEW QUESTIONS

		Not at all 0	1	2	3	4	5	6	Very Much 7
17)	Rate your sense of being in the virtual environment.	◯	◯	◯	◯	◯	◯	◯	◯

		At no Time 0	1	2	3	4	5	6	Most of the Time 7
18)	To what extent were there times during the experience when the virtual environment was real for you?	◯	◯	◯	◯	◯	◯	◯	◯

19) What is your current knowledge about computers?

◯ None
◯ Basic
◯ Intermediate
◯ Expert

20) How often do you play video games?

◯ Never
◯ Occasionally (once or twice a month)
◯ Frequently, but less than 50% of the days
◯ Frequently, 50% of the days or more

21) What is your current knowledge about VR?

◯ None
◯ Basic
◯ Intermediate
◯ Expert
